# Supplementary material for: Evidence supported by Mendelian randomization: impact on inflammatory factors in knee osteoarthritis
Source: Front Med (Lausanne). 2024 May 28;11:1382836. doi: 10.3389/fmed.2024.1382836 (PMC11165061; doi:10.3389/fmed.2024.1382836)
Supplement: Supplementary file 1 [file Data_Sheet_1.ZIP › data/GCST90274785_5e6/785.leaveoneout.pdf]

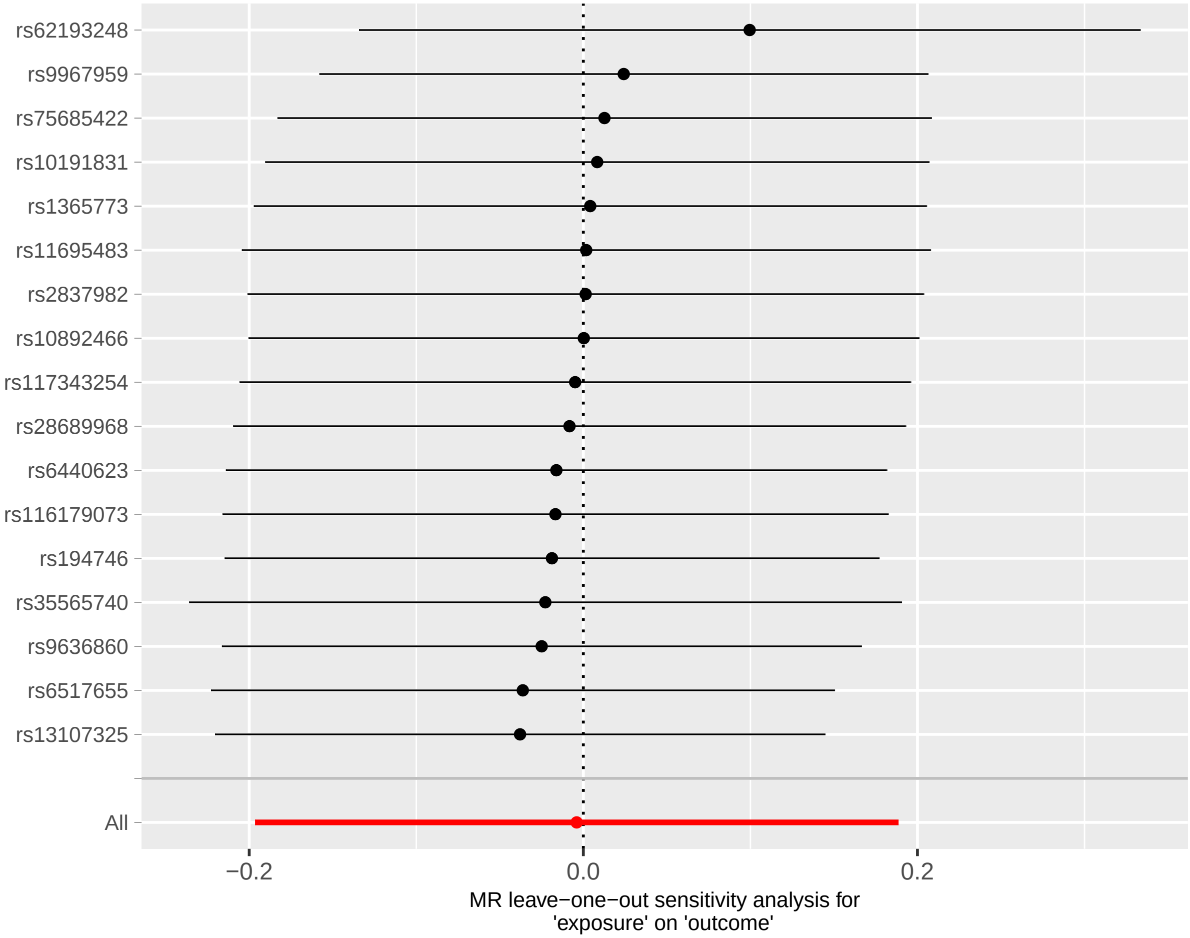
rs62193248
rs9967959
rs75685422
rs10191831
rs1365773
rs11695483
rs2837982
rs10892466
rs117343254
rs28689968
rs6440623
rs116179073
rs194746
rs35565740
rs9636860
rs6517655
rs13107325
All
−0.2
0.0
0.2
MR leave−one−out sensitivity analysis for
'exposure' on 'outcome'
